# Accumulation of Heavy Metals by Wild Mushrooms in Ibadan, Nigeria

**DOI:** 10.5696/2156-9614-7.16.26

**Published:** 2017-12-18

**Authors:** Chinatu Charity Ndimele, Prince Emeka Ndimele, Kanayo Stephen Chukwuka

**Affiliations:** 1. Department of Botany, Faculty of Science, University of Ibadan, Oyo State, Nigeria; 2. Department of Fisheries, Faculty of Science, Lagos State University, Ojo, Lagos, Nigeria

**Keywords:** mushroom, heavy metal, bioaccumulation

## Abstract

**Background.:**

Many companies in Nigeria generate industrial effluents, including heavy metals. These metals can be accumulated by biota such as mushrooms, which are then eaten by the populace.

**Objectives.:**

The present study investigates the metal content of wild mushrooms in order to educate the local population on the safety of their consumption.

**Methods.:**

Seven different species of wild mushrooms (Cortinarius melliolens, Chlorophyllum brunneum, Pleurotus florida, Volvariella speciosa, Cantharellus cibarius, Entoloma spp and Coprinus africana) growing in Ibadan, southwest Nigeria were analyzed for their heavy metal (copper (Cu), cadmium (Cd) and lead (Pb)) contents using atomic absorption spectrophotometry.

**Results.:**

The concentrations of the heavy metals in the mushrooms and substrate varied by site of collection. The highest concentrations of Cu (92.31±8.42 mg/kg), Pb (76.00±9.78 mg/kg) and Cd (92.45±12.34 mg/kg) were obtained in C. africana, P. florida and V. speciosa, respectively. The lowest contents of Cu (56.00±5.02 mg/kg), and Cd (67.92±5.89 mg/kg) were obtained from C. melliolens, while C. cibarius had the lowest concentration of Pb (40.00±3.56 mg/kg). The highest concentrations of Pb (20.40±3.43 mg/kg) and Cd (26.40±4.34 mg/kg) were obtained in the substrate of C. molybdites, while the lowest Pb (12.40±2.12 mg/kg) and Cd (18.00±3.90 mg/kg) occurred in V. speciosa and C. cibarius, respectively. The bioaccumulation factors of the mushroom species studied ranged from 2.84 – 14.60.

**Conclusions.:**

The present study found that heavy metal accumulation varied by species of mushroom, metal content of the substrate and the bioavailability of the metal in the mushroom. The level of metals in mushrooms in the present study was relatively high. Therefore, cultivation of mushrooms in heavy metal-free soil should be encouraged.

## Introduction

Malnutrition and starvation are two major nutritional problems facing millions of people in developing countries. Malnutrition has become an endemic disease in Nigeria because animal protein is in short supply as an increase in the livestock population is limited by viral disease, drought, scarcity and the high cost of feed. This situation has given rise to a considerable increase in demand for other sources of protein such as mushrooms. In addition, mushrooms are a better source of protein than most common foods.^1^

Populations in developing countries are rapidly increasing due to high levels of illiteracy and lack of access to birth control measures, among other factors. Consequently, there is a need for increased application of technologies to use natural resources to produce food, goods and services. This in turn leads to increased industrialization, which results in the release of industrial wastes such as heavy metals into the environment.

The distribution of heavy metals in different compartments of the ecosystem is regulated by physical and chemical processes (dilution, diffusion, precipitation and sorption), as well as other processes such as uptake and elimination.^2^ At low levels, metals such as copper (Cu), cobalt, zinc, iron and manganese are essential for enzymatic activity and many biological processes; however, these metals are toxic at high concentrations.^3^ Other metals such as cadmium (Cd), mercury (Hg) and lead (Pb) have no known essential role in living organisms and are toxic, even at low concentrations. Various studies have shown that metals such as Hg and Cd have toxic effects on aquatic and terrestrial organisms, altering physiological activities and biochemical parameters in their tissue and blood.^4^

Mushrooms are an important part of the local diet in southern Nigeria. Many vegetarians consume them as a source of protein in place of meat or fish. Their cultivation is not common, but edible species are mostly collected from the wild. They can also be a source of revenue, as people specialize in their collection and sale. The species of mushroom used in the present study include edible species which contribute to the protein intake of local people who may not be able to afford meat and fish.

Mushrooms are known to have the ability to accumulate heavy metals. These pollutants have detrimental effects not just on organisms in the environment, but also on humans through the food chain.^2^ Therefore, the aim of the present study was to investigate the metal content of local wild mushrooms with a view to educating locals on the safety of their consumption.

## Methods

Mushrooms samples were collected from different locations (highway, park, roadside and forest) within Ibadan city. The substrates on which the mushrooms were growing were also collected for heavy metal analysis. Mushrooms were rinsed in distilled water and oven dried at 80°C overnight.

### Determination of Heavy Metals in Mushrooms

Samples were ground using a milling machine after drying and 0.5 g of each sample was weighed into a digestion tube. The ground, dry homogenates were wet digested in a nitric-perchloric acid mixture. The samples were mixed and then brought to boil on a hot plate to the lowest volume possible (15 ml to 20 ml). The samples were made up to 50 ml with distilled water in a volumetric flask. Sample blanks were prepared in the laboratory in a similar manner to the field samples.^5^ The concentrations of heavy metals were determined by running samples in an Alpha-4 cathodeon atomic absorption spectrophotometer.^6^ Pb was determined at 283.3 nm, Cd at 218.8 nm and Cu at 324.7 nm. Standard solutions for system calibration and control of analytical accuracy were prepared by dilution of stock solutions (Merck, multi element standard). All specimens were run in batches that included blanks, a standard calibration curve, two spiked specimens, and one duplicate. In order to validate the method for accuracy and precision, dogfish muscle (DORM-2, National Research Council, Canada) was analyzed (n=6) as a certified reference material and the recovery (% mean recovery ± S.E.) was analyzed (n=6). The results showed good agreement between the certified and analytical values. The recovery was 97.6±4.1% for Cu, 98.32±3.60% for Cd and 96.8±4.1% for Pb. The precision of the analytical procedures, expressed as the relative standard deviation, ranged from 5 to 9%. The precision for the analysis of standard solution was better than 5%. All analyses were carried out in duplicate, and the results were expressed as the mean. Metal levels were expressed in mg/kg dry weight.

### Wet Digestion Materials for the Determination of Heavy Metals in Substrate (Soil)

Soil samples were dried to constant weight at room temperature and sieved with 2 mm mesh sieve to remove debris. Then 0.5 g of each soil sample was digested overnight with 0.1 N hydrochloric acid and filtered using No.1 Whatman filter paper.

The extracts were read on an atomic absorption spectrophotometer using the same wavelengths for the respective elements as were done previously for mushrooms.

Bioaccumulation factor is a measure of the ability of an organism (mushroom) to absorb and store the heavy metals from their substrates:

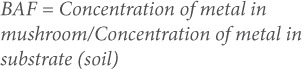



### Statistical Analysis

The data obtained were subjected to one-way analysis of variance (ANOVA) and where there was significant variation, Fisher's least significance difference was used to separate the means. In all cases, the level of significance was set at P>0.05.

## Results

The concentrations of the heavy metals varied significantly among the mushrooms. The highest Cu concentration was found in Coprinus africana (92.31±8.42 mg/kg), while the lowest value of 56.60±5.02 mg/kg was observed in Cortinarius melliolens (*Table 1*). The concentration of Cu in mushrooms (Chlorophyllum molybdites, Coprinus africana) collected on the road side was significantly (p<0.05) higher than those collected from park, lawn, forest and highway. The highest Pb concentration of 76.00±9.78 mg/kg was recorded in Pleurotus florida, while the lowest (40.00±3.56 mg/kg) was in Cantharellus cibarius. Mushroom samples (Pleurotus florida) collected from the park had Pb concentrations that were significantly (p<0.05) higher than samples collected from other sites.

**Table 1 —:**
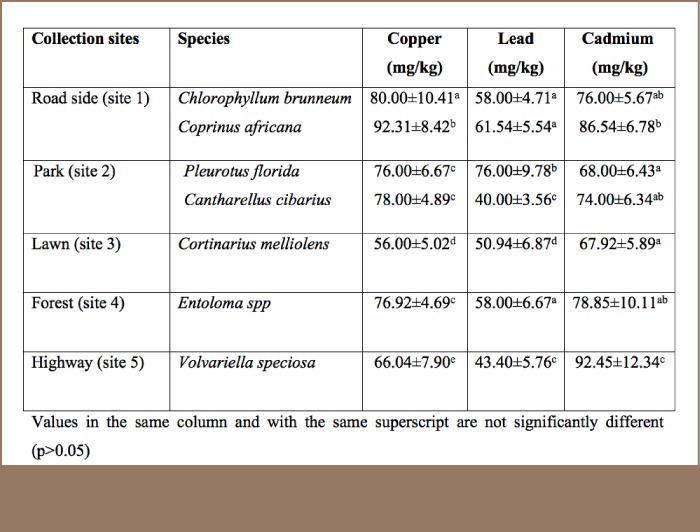
Concentrations of Copper, Lead and Cadmium in Wild Mushrooms at Five Collection Sites in Ibadan, Nigeria

Volvariella speciosa had the highest (92.45±12.34 mg/kg) Cd concentration among the mushroom species sampled. This was followed by C. africana (86.54±6.78 mg/kg), while the lowest concentration of Cd was recorded in C. melliolens (67.92±5.89 mg/kg). The mushroom sample from the highway had the highest Cd level, and this was significantly (p<0.05) higher than samples collected from other sites.

The concentrations of the metals in the substrates of the mushrooms were significantly (p<0.05) affected by site. The heavy metal contents of the mushroom substrates investigated varied from 12.40±2.12 mg/kg to 20.40±3.43 mg/kg for Pb, 16.00±3.43 mg/kg to 20.80±2.89 mg/kg for Cu, and 18.00±3.90 mg/kg to 26.40±4.34 mg/kg for Cd (*Table 2*).

**Table 2 —:**
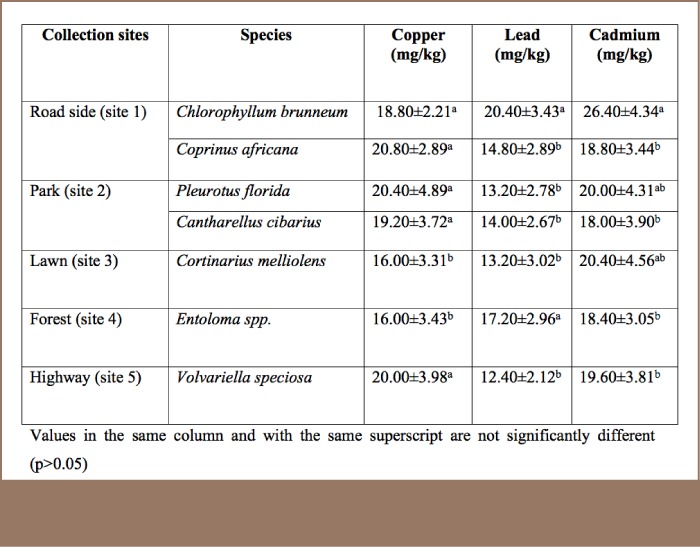
Concentrations of Copper, Lead and Cadmium in the Substrates of Wild Mushrooms at Five Collection Sites in Ibadan, Nigeria

The bioaccumulation factors ranged from 3.30 to 4.81 for Cu, 2.84 to 5.76 for Pb and 2.88 to 14.60 for Cd (*Table 3*).

**Table 3 —:**
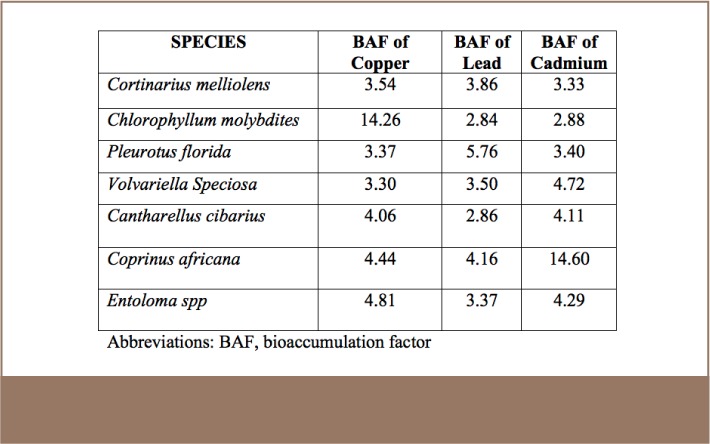
Bioaccumulation Factors of Copper, Lead and Cadmium in Wild Mushroom Samples Collected in Ibadan, Nigeria

## Discussion

The range of Cu concentrations (56.00±5.02 mg/kg to 92.31±8.42 mg/kg) obtained in the wild mushroom samples is similar to the range recorded in previous studies. Isiloglu et al.^7^ examined 66 samples of mushroom fruiting bodies representing seven species of edible mushrooms of Turkish origin and recorded a range of 26.30 mg/kg - 87.70 mg/kg. Demirbas^8^ determined the concentrations of twenty-one metals in 18 species of mushroom growing in the eastern Black Sea region and recorded a Cu concentration range of 5.11±0.67 mg/kg - 92.50±14.10 mg/kg. However, a relatively lower concentration (5.00 - 51.00 mg/kg) was reported by Tuzen et al.^9^ in twenty-four different species of uncultivated mushrooms of Turkish origin. The relatively high range recorded in this study and previous studies suggest that Cu content depends on the species of mushroom.^9^ It should also be noted that the difference in the highest and lowest concentrations of Cu and other metals is dependent on the pollution status of the environment where the mushroom is growing.

The lowest and highest values of Pb in wild mushroom species were 40.00±3.56 mg/kg in C. cibarius and 76.00±9.78 mg/kg in P. florida. The lowest and highest value of Pb was recorded in mushrooms collected from the park (University of Ibadan Botanical Garden). This further suggests that the heavy metal content of most mushrooms is species-dependent since the two mushroom species were collected from the same habitat. However, Kalac et al.^10^ reported that the amounts of trace elements are not only related to the particular mushroom species and collecting site, but also to other factors such as age of fruiting bodies, mycelium and distance from the pollution source. The last factor may have accounted for the difference in the Pb contents of the mushroom species recorded in the present study.

Cadmium levels in most edible species growing in an unpolluted (background) area are below 2 mg/kg d/w.^11^ However, the values recorded in the present study of 67.92±5.89 - 92.45±12.34 mg/kg are higher than this background value and the 5 mg/kg reported by Kalac and Svoboda^12^ in Boletus aestivalis, Leccinum scabrum, Calocybe gambosa, Armillaria mellea and Russula cyanoxantha, but lower than the 300 mg/kg in mushroom species analyzed by Schmitt and Meisch.^13^ The high level of Cd recorded in the present study is of particular concern because Cd is known as a principal toxic element, as it inhibits many life processes.^14^ However, the bioavailability of Cd in mushrooms is as low as 10%.^11^ This is due to various detoxification mechanisms which make Cd biologically unavailable. These mechanisms include intercellular detoxification (or specific Cd transport system) where a Cd-binding phosplastin binds Cd and renders it biologically unavailable.^8^ Another essential Cd-detoxification mechanism observed in the poisonous mycorrhizal mushroom Paxillus involutus involves Cd binding onto cell walls and accumulating in vacuolar compartments.^15^ Cadmium is accumulated mainly in the kidneys, spleen and liver and its level in blood serum increases considerably following mushroom consumption. Thus, Cd seems to be the most deleterious among the heavy metals found in mushrooms.^11^ Its acceptable daily or weekly intake may be easily reached by consumption of an accumulating mushroom species.^11^

The range of Cu content of the analyzed wild mushroom samples was 56.00±5.02 - 92.31±8.42 mg/kg, which is above the safe limit of 40 mg/kg set by the World Health Organization (WHO) in foods.^16^ Kalac reported that Cu accumulation in mushroom species usually varies from 20.0 to 100.0 mg/kg.^17^ According to the Food and Agriculture Organization (FAO)/WHO as reported by Kalac, tolerable weekly intakes (TWI) of Cd and Pb are 0.007 and 0.025 mg/kg body weight, respectively.^17^ The high values of Pb and Cd recorded in this study indicate that the TWI of these metals could be easily exceeded with consumption of a small quantity of these mushrooms.

The bioaccumulation factor of the seven mushroom species for the three heavy metals (Pb, Cu and Cd) investigated in this study are higher than the values reported in literature. Demirbas^18^ reported bioaccumulation factor values of less than one. Seeger^19^ reported a bioaccumulation factor of 0.01 - 0.1 for Pb. The relatively high bioaccumulation factor values obtained in this study suggest that the heavy metal source would most probably be the substrate and to a lesser extent the atmosphere due to the bioaccumulation and biomagnifications abilities of mushroom species. The surprising ability of some mushroom species to accumulate heavy metals prompted their testing as bioindicators for heavy metals.^11^ However, a review by Wondrastschek and Rider^20^ revealed that no mushroom species can be considered to be reliable indicators of environmental pollution caused by heavy metals.

## Conclusions

The present study found that the heavy metal accumulation by mushrooms depends on the species of mushroom, the metal content of the substrate and bioavailability of the metal. The heavy metal levels in mushrooms in the present study were relatively high. Therefore, cultivation of mushrooms in heavy metal-free soil should be encouraged.
